# RNA N6-Methyladenosine-Related Gene Contribute to Clinical Prognostic Impact on Patients With Liver Cancer

**DOI:** 10.3389/fgene.2020.00306

**Published:** 2020-04-23

**Authors:** Wei Wang, Bo Sun, Yang Xia, Shenghong Sun, Chiyi He

**Affiliations:** Department of Gastroenterology, Yijishan Hospital, Wannan Medical College, Wuhu, China

**Keywords:** liver cancer, m6A, ICGC, epigenetic modification, prognosis

## Abstract

Liver cancer (LC) is the fourth leading cause of cancer-related deaths worldwide. There is an urgent need to identify novel and reliable prognostic biomarkers for LC in order to improve patient outcomes. N6-methyladenosine (m6A) is the most common internal modification in eukaryotic mRNA and has been associated with various cancers, although its roles in the prognosis of LC remains to be elucidated. We analyzed the expression profiles of 15 m6A-related genes in the International Cancer Genome Consortium (ICGC) LIRI-JP dataset, and applied consensus clustering to stratify LC patients into two subgroups (Cluster 1 and Cluster 2). Cluster1 was significantly correlated to lower tumor stage and longer overall survival (OS). Gene set enrichment analysis showed that tumorigenic markers, including DNA repair, E2F targets, G2M checkpoint, and MYC targets V1, were enriched in Cluster2. We then constructed a prognostic risk model using three m6A-related genes that were identified as independent factors affecting OS. The nomogram based on the risk model score indicated good performance in predicting the 1-, 2- and 3-year survival of the LC patients. In conclusion, m6A-related genes are potential prognostic markers and therapeutic targets for LC.

## Introduction

Liver cancer (LC) is the fourth leading cause of cancer-related deaths worldwide (Villanueva, 2019). The etiology of LC differs geographically due to differences in the prevalence of risk factors (Jiang et al., 2019). For instance, chronic viral hepatitis infection is the most important risk factor in Asian countries, whereas non-viral factors are the major causative agents of LC in the Western countries (Yau et al., 2019). In East Asia, hepatitis B virus (HBV)- and hepatitis C virus (HCV)-related LC accounts for more than 80% of the cases (Liu et al., 2019a).

Liver tumorigenesis involves multiple steps with overlapping and interacting signaling pathways (Arzumanyan et al., 2013). However, the precise underlying mechanisms have not been completely elucidated so far. N6-methyladenosine (m6A), the most common internal post-transcriptional modification in eukaryotic mRNA, associates with many biological processes such as stress responses, stem cell differentiation, gametogenesis, and T Cell Homeostasis (Liu et al., 2019b; Zhou et al., 2019), and is mediated by factors that mainly include the “writers” (METTL3, METTL14, WTAP, RBM15, and ZC3H13), “readers” (YTHDC1, YTHDC2, YTHDF1, YTHDF2, YTHDF3, and HNRNPC), and “erasers” (FTO, ALKBH3, and ALKBH5) (Yang et al., 2018; Sun et al., 2019). Writers (m6A methyltransferase enzymes) and erasers (m6A demethylase enzymes) regulate the abundance, prevalence, and distribution of m6A, whereas readers (m6A binding proteins) modulate m6A modification-related mRNA processing, covering splicing, editing, localization, export, stability, translation, and decay (Zhou et al., 2019; Esteve-Puig et al., 2020). Recent reviews summarized that m6A-dependent mRNA regulation plays a crucial part in the development and progression of human cancers, such as HCC, acute myeloid leukemia (AML), glioblastoma, lung cancer, breast cancer, cervical cancer, and prostate cancers (Liu et al., 2019b; Esteve-Puig et al., 2020). Dysregulation of writers, readers, and erasers are pertinent to tumor initiation and progression, metastasis, and cancer drug resistance (Esteve-Puig et al., 2020). For example, METTL3 and FTO promote pathogenesis through stabilizing specific sets of mRNAs in breast cancer and AML, respectively (Tan et al., 2015; Vu et al., 2017). Similarly, alterations of readers such as YTHDC2 and YTHDF2 are related to colorectal cancer and hepatic cancer, respectively (Tanabe et al., 2016; Chen et al., 2018).

Hepatocellular carcinoma (HCC) is classified into different subclasses based on pathological characteristics and/or transcriptomes (Hoshida et al., 2009; Calderaro et al., 2017), and no study has so far reported prognostic subclasses of LC based on the expression of m6A-related genes. Since the prognosis of LC patients depends on the etiology and the ethnicity and/or geographical region (Hashimoto et al., 2017; Villanueva, 2019), and as East Asia has the highest incidence of LC (Bray et al., 2018), we therefore analyzed the m6A profile in an East Asian LC cohort (LIRI-JP dataset) from the International Cancer Genome Consortium (ICGC) database. The aim of this study was to determine the prognostic value of the m6A-related gene signature in LC.

## Materials and Methods

### Datasets

The RNA sequencing data and corresponding clinicopathological information of LC patients were extracted from the ICGC (LIRI-JP dataset^1^) and The Cancer Genome Atlas (TCGA, LIHC dataset^2^) databases in May 2019. The gene expression data from TCGA was estimated as Transcripts Per Kilobase of the exon model per Million mapped reads (TPM). In the LIRI-JP dataset, the clinical stages of the patients were classified as per the Stage of Liver Cancer Study Group of Japan (LCSGJ) guidelines. The simple somatic mutation data was also retrieved for calculating the tumor mutation burden (TMB). The data of non-solid tissues and non-primary tumors, and of samples lacking sufficient clinical information were excluded. In case two or more samples were derived from the same patient, the mean value was used for analysis. Finally, 231 LC patients and 199 healthy controls from the LIRI-JP dataset, and 370 LC patients from the LIHC dataset were selected. The clinicopathological data of all patients are summarized in Supplementary Table S1.

### Bioinformatics Analysis

Fifteen m6A-related genes were extracted from the LIRI-JP dataset (Supplementary Table S2). We analyzed the expression of 15 m6A-related genes in LC patients and normal tissue using the Limma package. LC patients were then clustered into different subgroups using the “Consensus Cluster Plus” package. In order to functionally annotate differentially expressed genes (DEGs) in different subgroups, Gene Ontology (GO) and Kyoto Encyclopedia of Genes and Genomes (KEGG) pathway enrichment analyses were conducted using the “clusterProfiler” package (Yu et al., 2012), and the Gene set variation analysis (GSVA) R package was used to analyze significant differences between the subgroups (Hanzelmann et al., 2013). Gene set enrichment analysis (GSEA) was used to identify the hallmarks of tumor sets in different LC subgroups (Liberzon et al., 2015).

The prognostic values of the m6A-related gene were determined by univariate Cox regression analysis in the LIRI-JP dataset in terms of hazard ratio (HR) and 95% confidence interval (CI). Six prognostic relevant genes (*P* < 0.05) were then used for the multivariable Cox analysis by step-wise forward and backward selection approaches as well as the smallest Akaike information criterion (AIC). Finally, a risk model was constructed using three genes, and the risk score (designated as riskScore) was calculated for each patient in the LIRI-JP and LIHC dataset using the formula: riskScore=Coef_*gene1*_×Exp_*gene1*_+Coef_*gene2*_×Exp_*gene2*_+Coef_*gene3*_×Exp_*gene3*_, where Coef is the coefficient and Exp is the gene expression value. The clinicopathological factors and riskScore were used as variates in the univariate and multivariate Cox proportional hazards (PH) regression analyses to determine the independent predictive factors of overall survival (OS) in both datasets. A nomogram for 1-, 2-, and 3-year OS was then constructed based on the independent predictive factors, and its predictive performance was evaluated by C-index (Harrell et al., 1996). The calibration curve of the nomogram was used to assess the congruency between the predicted and actual survival. Bootstraps with 1,000 resamples were used to quantify model overfit, and a decision curve analysis (DCA) was made to evaluate the clinical efficacy (Vickers et al., 2008). The prediction power of the distinct parameters was determined using the area under receiver operating characteristic (ROC) curve (AUC) values.

### Statistics

The expression level of 15 genes in the LC patients and controls was analyzed using the Wilcoxon rank sum test. The correlation between genes was determined by Pearson’s analysis. Patients were divided into different groups by consensus analysis or riskScore (median value as the cutoff), and the distribution of clinical parameters between the subgroups was determined by Fisher’s exact test. The OS of LC patients in the different subgroups was analyzed by the Kaplan–Meier method and compared with the log-rank test. All statistical analyses were performed by R v3.6.0^3^.

## Results

### Differentially Expressed m6A-Related Genes Classify Liver Cancer Patients Into Distinct Clinical Clusters

**FIGURE 1:**
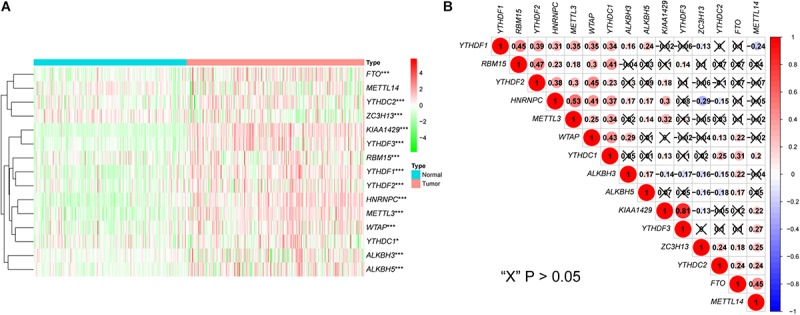
Expression and correlation of m6A-related genes in liver cancer. **(A)** The expression levels of 15 m6A-related genes in liver cancer (Normal = 199, Tumor = 231). The heatmap shows the fold changes, with green indicates down-regulated genes, and red indicates up-regulated genes. **(B)** Pearson’s correlation analysis of the 15 m6A-related genes. Blue indicates significant negative correlation and red indicates positive. *P < 0.05, **P < 0.01, ***P < 0.001.

**FIGURE 2:**
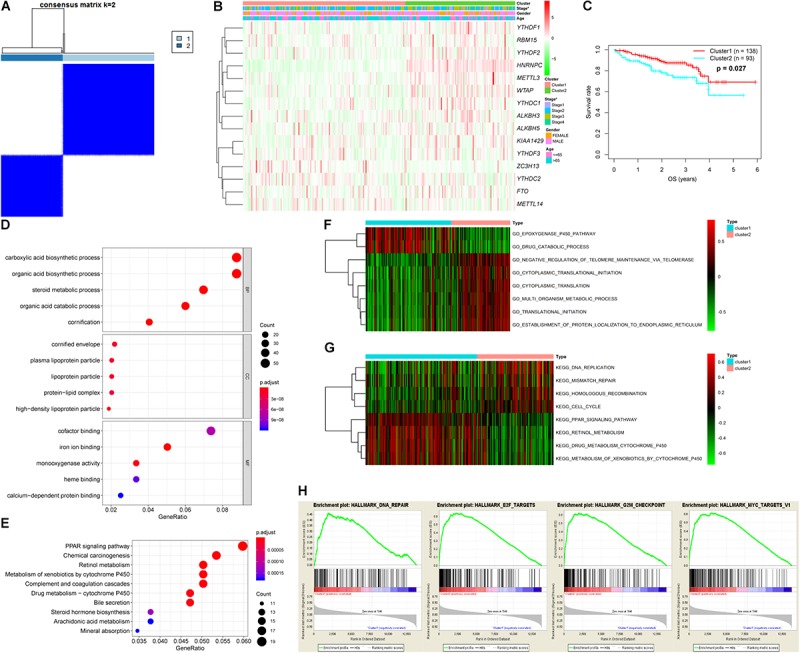
Differential tumor stage and overall survival and functional annotation of liver cancer in Cluster 1 (*n* = 138) and Cluster 2 (*n* = 93) subgroups. **(A)** Consensus clustering matrix for *k* = 2. **(B)** Heatmap and clinicopathologic features of the two clusters defined by the m6A-related genes consensus expression. Green and red in the heat map indicate down-regulated and up-regulated genes, respectively. **(C)** Kaplan–Meier overall survival curves for liver cancer patients in LIRI-JP dataset. **(D,E)** Functional annotation of differentially expressed genes between Cluster 1 and Cluster 2 subgroups by GO terms **(D)** and KEGG pathway **(E)**. **(F,G)** GO terms **(F)** and KEGG pathway **(G)** significantly enriched in GSVA. **(H)** Genes with higher expression in Cluster 2 subgroup were enriched for hallmarks of tumor sets by GSEA.

Analysis of the expression patterns of m6A-related genes in the LIRI-JP dataset identified 14 DEGs in this study, including *KIAA1429*, *HNRNPC*, *METTL3*, *YTHDF3*, *YTHDF1*, *FTO*, *WTAP*, *YTHDF2*, *ALKBH5*, *ZC3H13*, *YTHDC2*, *ALKBH3*, *RBM15*, and *YTHDC1*. Among these DEGs, 13 genes were up-regulated including *KIAA1429*, *HNRNPC*, *METTL3*, *YTHDF3*, *YTHDF1*, *FTO*, *WTAP*, *YTHDF2*, *ALKBH5*, *YTHDC2*, *ALKBH3*, *RBM15*, and *YTHDC1*, while *ZC3H13* was down-regulated (Figure 1A and Supplementary Table S2). In addition, we analyzed the correlation among m6A-related genes. The KIAA1429 and YTHDF3 were highly correlated with each other, both of them were positively correlated with METTL14 and negatively correlated with ALKBH3, respectively. For “readers,” YTHDF1 was positively correlated with YTHDF2, HNRNPC, and YTHDC1. For “writers,” WTAP was positively correlated with RBM15, METTL3, and YTHDC1. For “erasers,” FTO was positively correlated with ALKBH3 and ZC3H13, whereas ALKBH3, and ZC3H13 were negatively correlated with each other (Figure 1B). According to the consensus clustering analysis, the LC patients were divided into Cluster 1 (n = 138) and Cluster 2 (*n* = 93) (Figure 2A and Supplementary Figure S1). Then, we compared the clinical features of these two Clusters. Cluster 1 was significantly correlated with lower tumor stage (*P* < 0.05), but not with gender and age (Figure 2B). Figure 2C showed that prolonged overall survival (OS) in patients with Cluster 1, and the 3-year survival rates of Cluster 1 and Cluster 2 subgroups were 87.3 and 73.8%, respectively (*P* < 0.05). In addition, *YTHDF2* levels were significantly lower in stage 1 and 2 tumors compared to that in stages 3 and 4 (*P* < 0.01), while similar trends were not observed with *METTL3* and *YTHDC2* (Supplementary Figure S2). Then, we identified 761 DEGs between Cluster 1 and Cluster 2 with | fold change| > 1 and FDR < 0.05 as the criteria. GO and KEGG pathway analyses showed that these DEGs mainly participated in malignancy-related pathways, including PPAR signaling pathway, retinol metabolism, chemical carcinogenesis, and xenobiotics- and drug metabolism-related cytochrome P450 (Figures 2D,E). GSVA resulted in similar findings (Figures 2F,G). Furthermore, GSEA indicated that hallmarks of tumor sets were remarkably enriched in DNA repair (NES = 1.74, normalized *P* < 0.05), E2F targets (NES = 1.91, normalized *P* < 0.05), G2M checkpoint (NES = 1.91, normalized *P* < 0.05), and MYC targets V1 (NES = 1.82, normalized *P* < 0.05) in the Cluster 2 subgroup (Figure 2H).

### Three m6A-Related Genes Form a Prognostic Risk Signature in Liver Cancer

**FIGURE 3:**
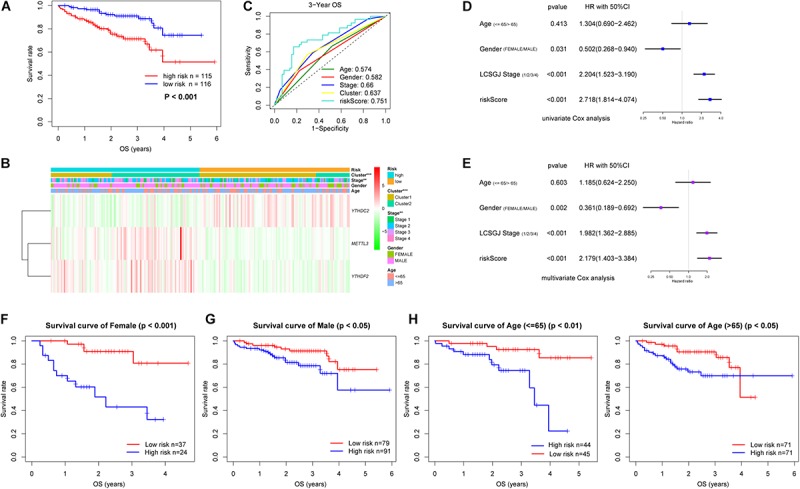
Risk signature with three m6A-related genes in LIRI-JP dataset. **(A)** Kaplan–Meier overall survival curves for liver cancer patients classified into high and low risk groups based on the riskScore. **(B)** The differential clinicopathological features was compared between the high and low risk groups. Green and red in the heat map indicate down-regulated and up-regulated genes, respectively. **(C)** ROC curves displayed the predictive power of the riskScore, age, gender, tumor stage and cluster for the 3-year survival rate. **(D)** Univariate and **(E)** multivariate Cox regression analyses of the association between clinicopathological factors and overall survival. **(F–I)** Prognostic value of the riskScore stratified by **(F,G)** gender and **(H,I)** age. LCSGJ: Liver Cancer Study Group of Japan, **P < 0.01, ***P < 0.001.

Six m6A-related genes significantly correlated with OS by Univariate Cox analysis (*P* < 0.05), of which *METTL3*, *YTHDC2*, and *YTHDF2* were identified as independent predictors of OS and the coefficients were obtained by the multivariate analysis (Table 1). A risk model was constructed using these genes, and the riskScore was calculated for LC patients. Using the median riskScore as the cutoff value, we classified the LC patients into the high and low risk groups and observed poorer OS in the former (*P* < 0.001; Figure 3A). In addition, the risk subgroups differed significantly in terms of tumor stage (*P* < 0.01) and gene cluster (*P* < 0.001) (Figure 3B), but not age and gender in the LIRI-JP dataset (Supplementary Figure S3). The AUC values showed a better predictive ability of the riskScore for 3-year OS compared to the aforementioned parameters (Figure 3C). The multivariate analysis confirmed that riskScore, gender and stage were independent prognostic factors for the OS (stage and riskScore, *P* < 0.001; gender, *P* < 0.01) (Figures 3D,E). Furthermore, the female and Stage 4 LC patients had poorer prognosis compared to the male patients and those at other tumor stages, respectively (Supplementary Figures S4A–C). Interestingly, patients in the low risk subgroup stratified further by gender (female, *P* < 0.001; male, *P* < 0.05) or age (≤65, *P* < 0.01; >65, *P* < 0.05) had relatively longer OS compared to those in the high risk subgroup (Figures 3F–I), whereas the tumor stage was not affected by the riskScore (Supplementary Figures S4D–G).

**TABLE 1:** Univariate and multivariate analyses of fifteen m6A-related genes in LIRI-JP dataset.

**Gene**	**Univariate analysis**			**Multivariate analysis**			
	**HR**	**95% CI**	***P***	**Coef**	**HR**	**95% CI**	***P***
*ALKBH5*	1.06	1.01–1.11	0.017	–	–	–	–
*FTO*	1.04	0.90–1.20	0.641	–	–	–	–
*HNRNPC*	1.02	1.01–1.03	0.005	–	–	–	–
*KIAA1429*	1.07	1.00–1.15	0.066	–	–	–	–
*METTL14*	0.92	0.81–1.05	0.211	–	–	–	–
*METTL3*	1.05	1.03–1.08	9.75E-05	0.039	1.04	1.01–1.07	0.004
*RBM15*	1.24	0.99–1.55	0.066	–	–	–	–
*WTAP*	1.03	1.00–1.07	0.101	–	–	–	–
*YTHDC1*	1.01	0.89–1.13	0.929	–	–	–	–
*YTHDC2*	0.81	0.70–0.95	0.009	-0.176	0.84	0.72–0.98	0.024
*YTHDF1*	1.05	1.00–1.10	0.034	–	–	–	–
*YTHDF2*	1.07	1.02–1.12	0.005	0.035	1.04	0.99–1.09	0.142
*ZC3H13*	0.98	0.94–1.01	0.155	–	–	–	–
*ALKBH3*	0.96	0.87–1.06	0.436	–	–	–	–
*YTHDF3*	1.00	0.96–1.05	0.939	–	–	–	–

### Validation of the Risk Signature in TCGA Cohort

**FIGURE 4:**
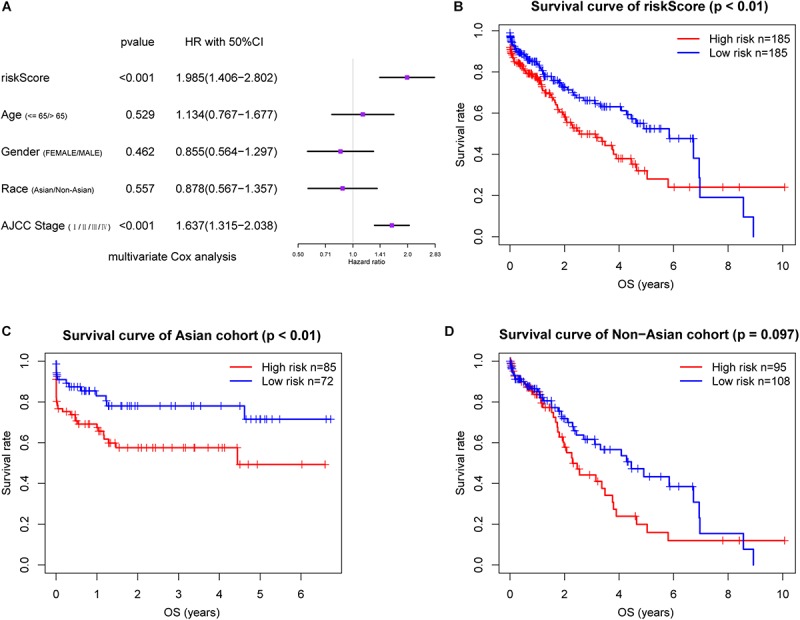
Validation of the risk signature in TCGA cohort. **(A)** Multivariate Cox regression analysis of the association between clinicopathological factors and overall survival. Prognostic value of the riskScore in TCGA cohort **(B)** and stratified by **(C,D)** race. AJCC, American Joint Committee on Cancer.

In the TCGA dataset, *METTL3*, *YTHDC2*, and *YTHDF2* were also significantly upregulated in the LC patients relative to the controls (Supplementary Table S2), and the riskScore was an independent prognostic factor for OS in this cohort (Figure 4A). We also stratified these patients into the high and low riskScore groups as with the LIRI-JP cohort, and observed significantly poorer prognosis in the former (*P* < 0.01) (Figure 4B). Furthermore, the low riskScore group had longer OS compared to the high riskScore group in the Asian cohort (*P* < 0.01) (Figure 4C). Although there was no significant difference, a trend of better survival in the low risk group was observed in the non-Asian cohort (Figure 4D). The AUC values showed that the riskScore had moderate predictive ability for 1-, 2-, and 3-year OS in the TCGA dataset (Supplementary Figure S5A), and the AUC values in the Asian cohort were higher than those in the non-Asian cohort (Supplementary Figures S5B,C).

### Construction and Validation of Nomogram

**FIGURE 5:**
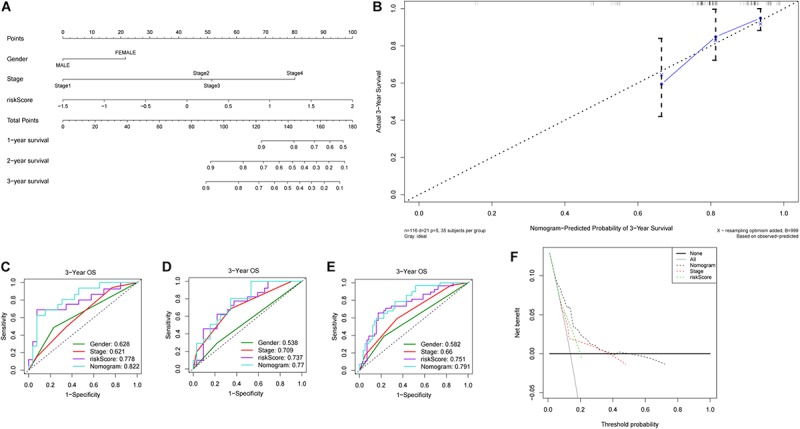
Construction and validation of nomogram. **(A)** Nomogram predicting 1-, 2- and 3-year OS of patients with liver cancer. **(B)** Calibration plot for predicting patient OS at 3-year. ROC curves of the nomogram and clinicopathological factors for 3-year OS prediction in the **(C)** training cohort, **(D)** validation cohort and **(E)** entire cohort. **(F)** Decision curves of the nomogram, tumor stage and riskScore for predicting OS.

The 231 LC patients in the LIRI-JP dataset were arbitrarily separated into the training (*n* = 116) and validation cohort (*n* = 115) with a 5:5 split ratio (seeds = 100). In the training cohort, all independent prognostic factors were included in the predictive nomogram for OS (Figure 5A), and the points for each predictor are listed in Supplementary Table S3. The calibration curves indicated good congruency between the predicted and observed 3-year OS (Figure 5B). The Harrell’s concordance-index (C-index) and 3-year AUC value of the nomogram were 0.797 and 0.822, respectively, which were higher compared to that of the riskScore, gender, or tumor stage (Figure 5C and Table 2). Similar outcomes were obtained in the validation as well as the entire cohort (Figures 5D,E and Table 2). In addition, DCA curves showed a greater threshold of the nomogram compared to the riskScore or tumor stage (Figure 5F), indicating that the nomogram has greater discriminatory capacity and accuracy for predicting survival compared to the other factors.

**TABLE 2:** Comparison of C-index between the nomogram and other parameters in LIRI-JP cohort.

	**C-index (95% confidence interval)**		
	**Training cohort**	**Validation cohort**	**Entire cohort**
Nomogram	0.797 (0.715–0.879)	0.800 (0.718–0.882)	0.791 (0.732–0.850)
riskScore	0.709 (0.599–0.819)	0.714 (0.596–0.832)	0.706 (0.626–0.786)
Stage	0.667 (0.549–0.785)	0.729 (0.637–0.821)	0.699 (0.625–0.773)
Gender	0.591 (0.475–0.706)	0.566 (0.454–0.678)	0.579 (0.499–0.659)

### The m6A-Related Nomogram Has High Predictive Power

**FIGURE 6:**
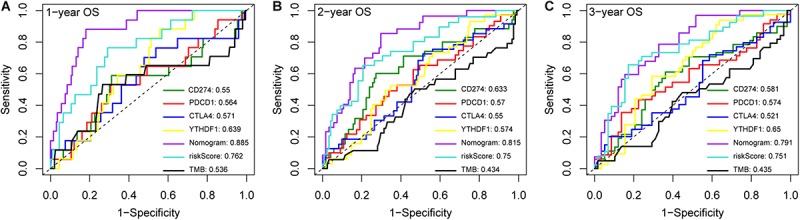
Compare the AUC values of the nomogram with different biomarkers. ROC curves of the nomogram and different biomarkers for **(A)** 1-, **(B)** 2- and **(C)** 3-year overall survival prediction in LIRI-JP cohort.

**FIGURE 7:**
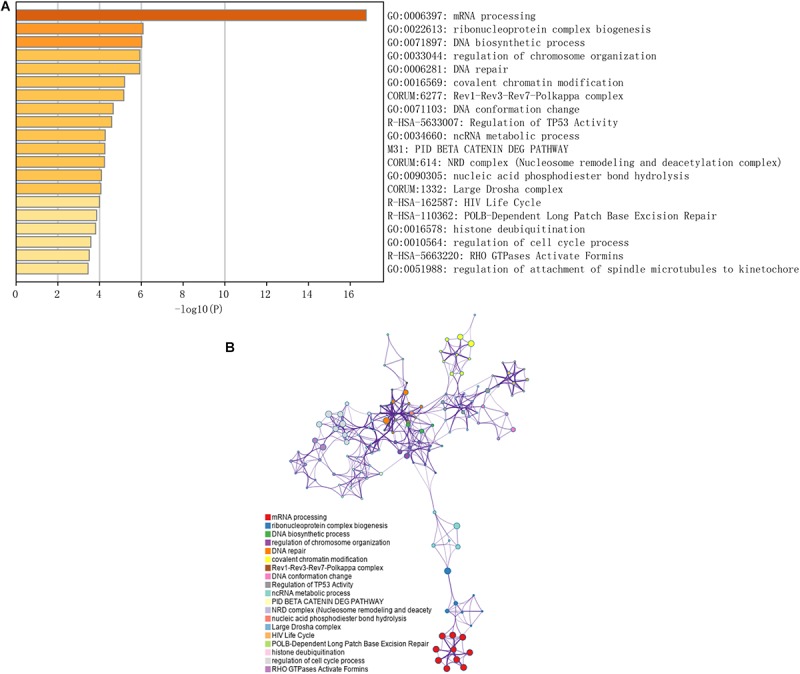
Functional prediction of three m6A-related genes involved in the risk signature. **(A)** Significantly enriched pathways of the three genes and their coexpressed genes. **(B)** The map of functional enriched pathways. Each node represents a GO term. Node size represents the number of gene in the pathway. Different colors represent different pathways.

Immune checkpoint proteins including the programmed cell death protein 1 (PD-1/PDCD1), programmed death-ligand-1 (PD-L1/CD274), and cytotoxic T-lymphocyte associated antigen 4 (CTLA-4) are established prognostic markers for LC patients (Cariani and Missale, 2019; El Dika et al., 2019; Johnston and Khakoo, 2019). Recent studies showed that tumor mutation burden (TMB) is also significantly associated with the susceptibility to anti-tumor immunotherapy, and a higher TMB indicates better prognosis in many cancer types (Peng et al., 2019; Wang and Li, 2019). The m6A-related gene *YTHDF1* was also closely related to the prognosis of HCC in the TCGA dataset in a previous study (Zhao et al., 2018). We compared the AUC values of our established nomogram with these biomarkers, and found that the predictive power of the nomogram was superior for 1-, 2-, and 3-year OS in the LIRI-JP dataset (Figure 6). Finally, pathway enrichment analysis by Metascape^4^ indicated that *METTL3*, *YTHDC2*, and *YTHDF2* and their 100 most strongly correlated co-expressed genes were enriched for functions like mRNA processing, DNA repair, covalent chromatin modification, and regulation of the cell cycle, which are closely involved in tumorigenesis (Figure 7).

## Discussion

Although numerous genes and non-coding RNAs associated with LC progression have been identified in recent years (Tsuei et al., 2004; Yuan et al., 2014; Chua et al., 2015; Li et al., 2016; Mattu et al., 2016; Xiao et al., 2016; Zhang et al., 2016; Liu et al., 2017; Zhao et al., 2018; Zhou et al., 2019), the prognosis of the patients remains disappointing. Therefore, it is imperative to identify novel and reliable prognostic biomarkers or models in order to improve the clinical outcomes of LC patients. LC is a highly heterogenous cancer, and patient prognosis depends significantly on the geographical region and etiology. Chronic infection with the hepatitis virus is a major risk factor of LC in East Asia, whereas alcohol consumption and non-alcoholic fatty liver disease are the main causes in Western countries. We analyzed the gene expression data of East Asian LC patients from the ICGC LIRI-JP dataset in order to determine the prognostic potential of m6A-related genes in LC. We found that six m6A-related genes were significantly associated with the malignant progression and prognosis of LC, and a risk signature consisting of three of these genes was predictive of the prognosis.

We used consensus clustering to stratify the patients into two subgroups based on the expression of m6A-related genes, which showed significant differences in OS and the enriched pathways associated with tumor development and progression. The prognostic risk model also stratified the patients in the LIRI-JP cohort into two groups based on the 3-gene riskScore, which showed greater predictive performance compared to single clinical indicators. Multivariate Cox analysis revealed that the riskScore was an independent prognostic factor for LC in the LIRI-JP and LIHC datasets. The nomogram, constructed using the riskScore and clinicopathological features, further increased the predictive power for OS compared to the riskScore, immunotherapy-related genes, or TMB alone. Interestingly, in the TCGA dataset, the riskScore was able to make a distinction for the OS in the Asian cohort, but not in the non-Asian cohort. This difference may be due to the fact that risk factors for LC differ across ethnicities.

The three genes (*YTHDC2*, *YTHDF2*, and *METTL3*) incorporated in the prognosis risk model were upregulated in the LC patients in both LIRI-JP and LIHC datasets, which are similar to those of previous studies (Yuan et al., 2014; Chen et al., 2018). *YTHDF2* and *METTL3* have previously been reported as tumor suppressors in HCC, and as oncogenes in pancreatic cancer and acute myelocytic leukemia (Cui et al., 2017; Wang et al., 2017; Zhong et al., 2019). Chen et al. (2018) demonstrated that overexpression of *METTL3* in HCC patients have poor prognosis. Further, knockout of *METTL3* suppresses HCC tumorigenicity and lung metastasis by modulation of cytokine signaling 2 through a *YTHDF2*-dependent mechanism (Chen et al., 2018). Zhong et al. (2019) demonstrated that *YTHDF2* acts as an HCC suppressor *via* promoting the degradation of epidermal growth factor receptor mRNA. Hou et al. (2019) reported that a high expression of *YTHDF2* gives rise to a better prognosis of HCC patients, and represses tumor growth and angiogenesis by degradation of interleukin 11 and serpin family E member 2 mRNAs. Tanabe et al. (2014) reported that *YTHDC2* acts as a tumor suppressor in the LC cell line by perhaps recruiting c-Jun and activating transcription factor 2 to the *YTHDC2* promoter. The above three m6A-related genes may affect HCC growth and metastasis by regulating the stability of multiple target genes.

Recent studies showed that m6A-related genes could be potential prognostic markers for predicting patient survival in a variety of cancers. These genes significantly correlated and interacted with each other which indicated that the cross-talk exists among the m6A-related genes (Li et al., 2019). Because of a complex reciprocal regulatory relationship among the m6A-related genes, it seems necessary to analyze prognostic and predictive values using a signature comprised of multiple m6A-related genes in patients with distinct tumor types. Kandimalla et al. (2019) reported that a gene expression signature consisted of seven m6A-related regulators characterized as a robust prognostic and predictive signature in 13 human cancers including HCC (relapse-free survival). This study offered a landscape of the biological and clinical characteristics pertaining to m6A machinery in tumor patients (Kandimalla et al., 2019). However, the external validation cohort was applied to colorectal cancer, gastric cancer, breast cancer, and ovarian cancer, but not to HCC for survival analysis. In our study, we successfully established a prognostic signature comprised of three m6A-related genes for predicting survival of HCC patient, using an additional RNA-seq dataset as external validation avoiding biased results to some extent.

There were some limitations in this study. First, an additional LC patient cohort for a prognostic study was needed to validate the predictive power of our prognostic signature in the future. Second, experimental studies that focus on the molecular mechanisms remain necessary to investigate the functions of these m6A-related genes in LC.

In summary, m6A-related genes have a prognostic value in LC, and the constructed riskScore can identify patients who are high risk and can enable individualized therapy. Our findings have to be validated in larger cohorts, and further studies are also needed to elucidate the mechanism of these m6A-related genes in LC.

## Data Availability Statement

The data used to support the results of this study are from the public databases ICGC (International Cancer Genome Consortium, https://icgc.org/) and TCGA (The Cancer Genome Atlas, https://cancergenome.nih.gov/).

## Ethics Statement

Ethical review and approval was not required for the study on human participants in accordance with the local legislation and institutional requirements. Written informed consent for participation was not required for this study in accordance with the national legislation and the institutional requirements.

## Author Contributions

CH designed the research study. WW, BS, YX, and SS analyzed the data and performed the data analysis. WW wrote the manuscript and interpreted the data. CH helped to revise the manuscript. All authors read and approved the final manuscript.

## Conflict of Interest

The authors declare that the research was conducted in the absence of any commercial or financial relationships that could be construed as a potential conflict of interest.

## Footnotes

1 https://icgc.org/

2 http://cancergenome.nih.gov/

3 https://www.r-project.org/

4 http://metascape.org/
