# Supplementary material for: RNA N6-Methyladenosine-Related Gene Contribute to Clinical Prognostic Impact on Patients With Liver Cancer
Source: Front Genet. 2020 Apr 23;11:306. doi: 10.3389/fgene.2020.00306 (PMC7190981; doi:10.3389/fgene.2020.00306)
Supplement: Supplementary file 1 [file Data_Sheet_1.pdf]

frontiers

Supplementary Material

Supplementary Figures

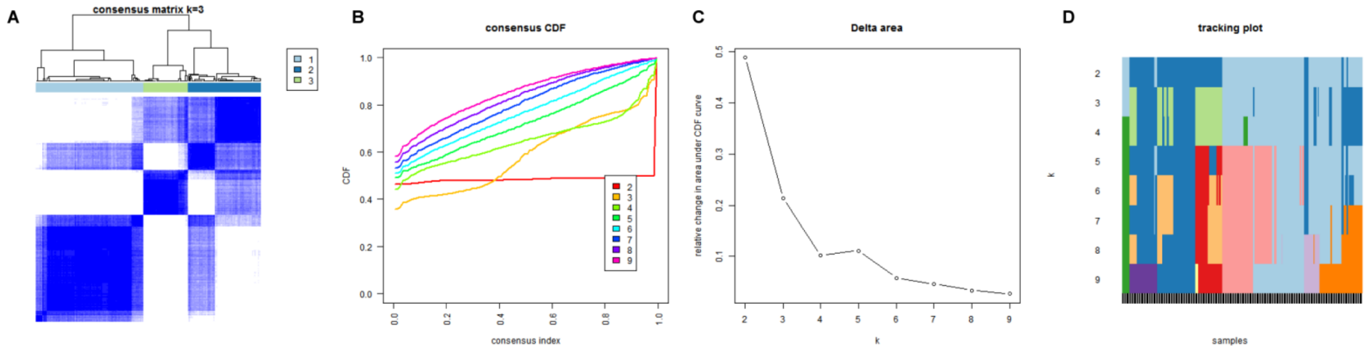

Figure S1. Identification of consensus clusters by m6A-related genes. **(A)** Consensus clustering matrix for k = 3. **(B)** Consensus clustering cumulative distribution function (CDF) for k = 2 to 10. **(C)** Relative change in area under CDF curve for k = 2 to 10. **(D)** The tracking plot for k=2 to k=10.

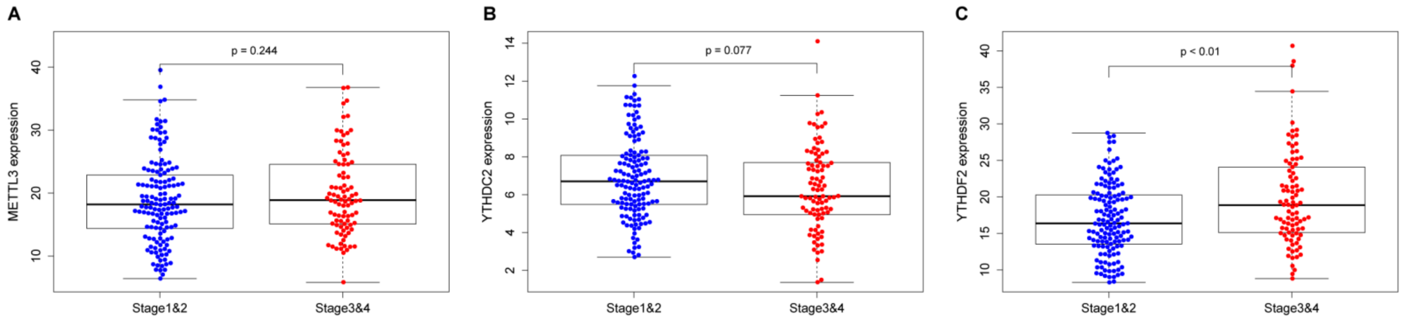

Figure S2. Expression levels of **(A)** *METTL3*, **(B)** *YTHDC2* and **(C)** *YTHDF2* in liver cancer patients with different tumor stages.

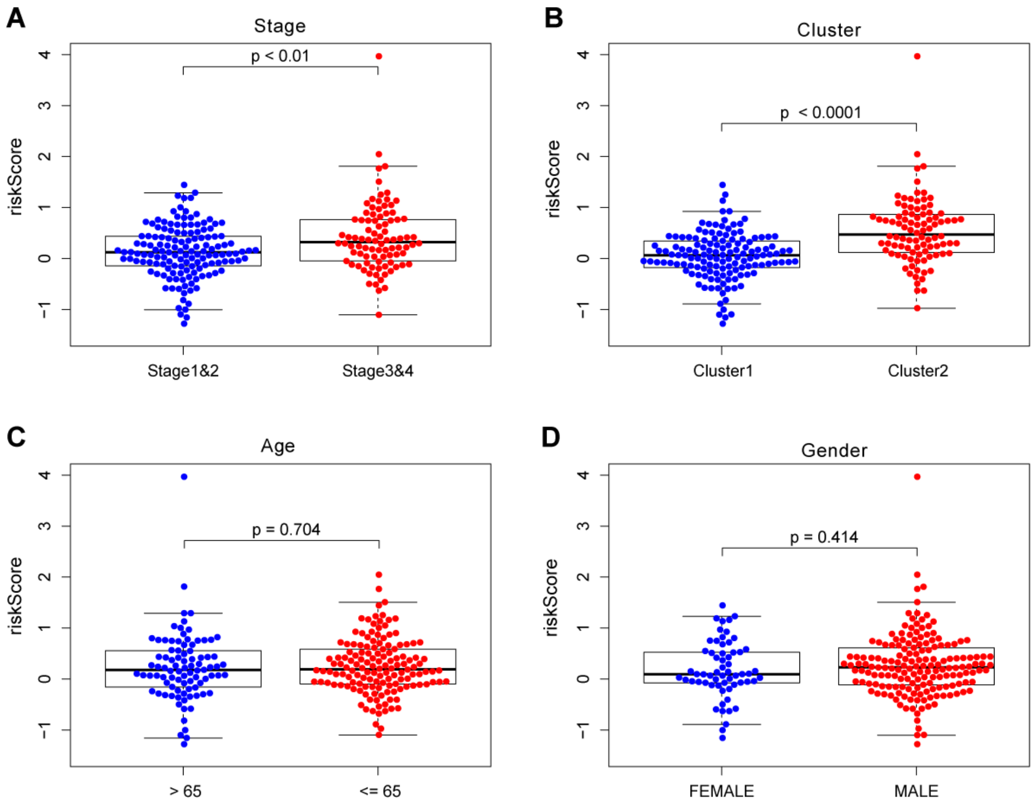

Figure S3. Relationship between the riskScore and different clinicopathological factors in the LIRI-JP dataset. **(A—D)** Distribution of riskScore stratified by **(A)** tumor stage, **(B)** cluster, **(C)** age and **(D)** gender.

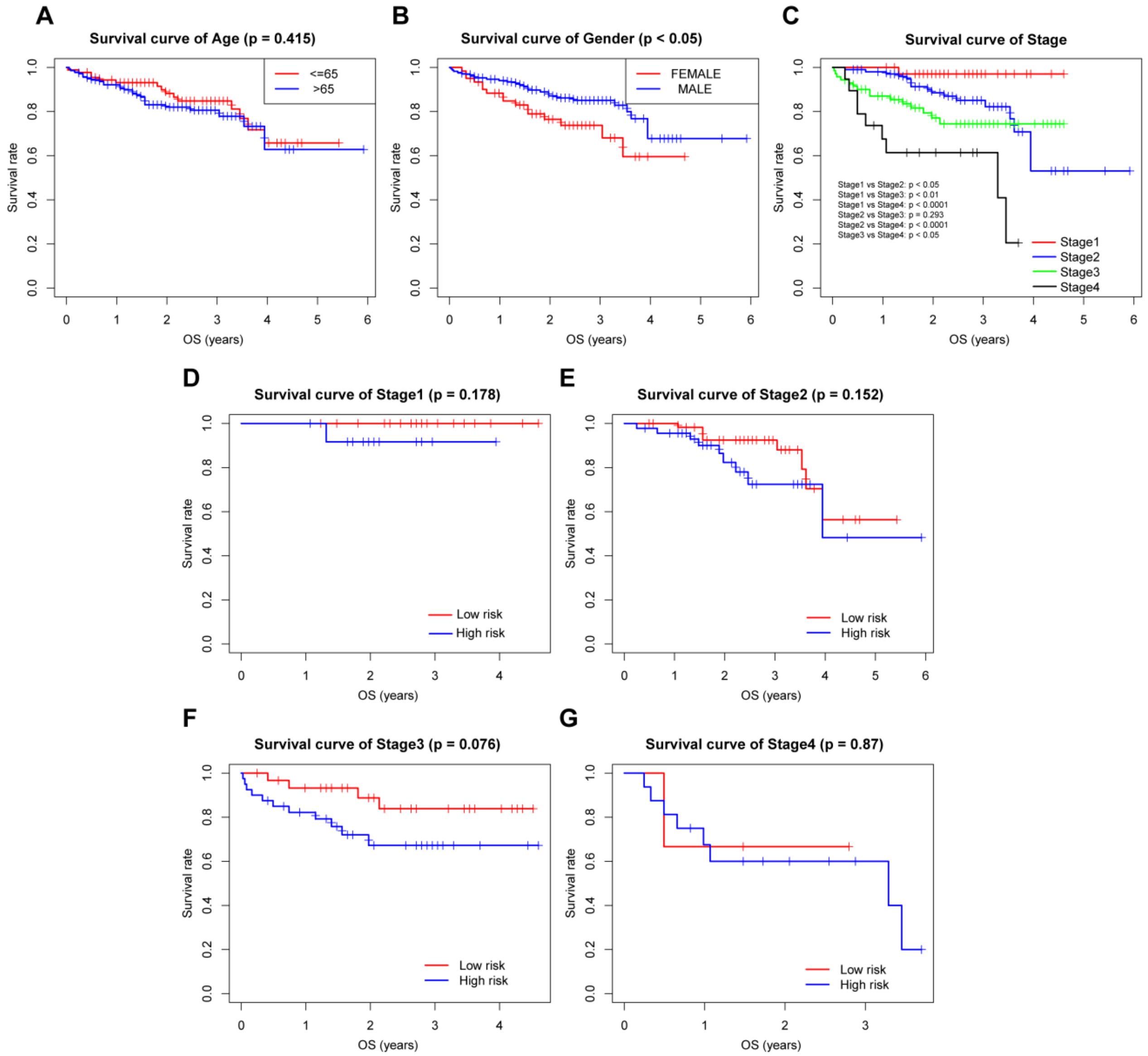

Figure S4. Prognostic value of liver cancer patients stratified by the different clinicopathological factors in the LIRI-JP dataset. **(A**—**C)** Kaplan-Meier overall survival curves for liver cancer patients with **(A)** age, **(B)** gender and **(C)** tumor stage. **(D**—**G)** Kaplan-Meier overall survival curves for different tumor stage with riskScore.

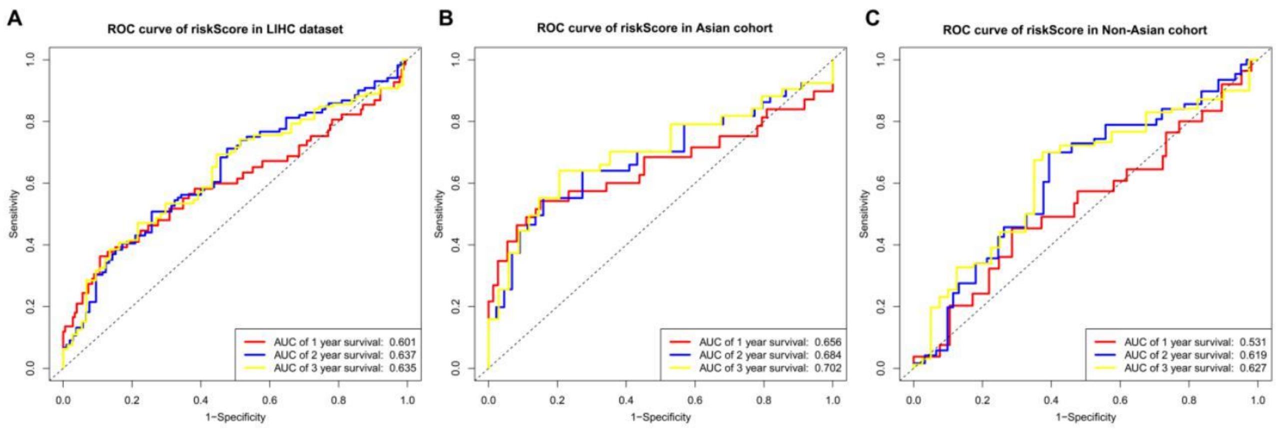

Figure S5. Predictive ability of the riskScore for overall survival in TCGA cohort. Overall survival predictive ability of the riskScore in **(A)** LIHC dataset, **(B)** Asian cohort and **(C)** Non-Asian cohort.
